# Use of Expired Drugs: Patients Benefits versus Industry Interest

**DOI:** 10.31662/jmaj.2022-0209

**Published:** 2023-11-16

**Authors:** Anis Arioua, David Shaw

**Affiliations:** 1 Institute for Biomedical Ethics, University of Basel, Basel, Switzerland

**Keywords:** expiry date, economy, pharmaceutical industry, ethics

## Abstract

The question of the use of expired medication is a constant debate of many years most especially in a difficult environment. In this research, a literature review and reported practice of some countries are used. Findings show that some medications, if properly stored, remain safe to use after the expiry date and this sometimes could be extended by many years. However, the pharmaceutical manufacturers have an interest in producing products with short shelf life. To dispose of expired ones is to generate a sale and therefore put the profit into the improvement and development of new products for the benefit of medicine.

This paper emphasizes the benefit of use of some drugs after expiration and the interest for the pharmaceutical industry to dispose of expired drugs and to shorten their shelf life.

## Introduction

The past decades have been marked by the rise of illnesses and chronic diseases, over prescription, changed prescriptions, canceled treatment, misuse of drugs, and excessive production of these drugs. Doctors may cancel prescribed medications if the patient experiences, for example, an allergy or any intolerance to the drug. They may also change to another medication more beneficial or more financially affordable for the patient. All this has increased both the consumption of pharmaceutical drugs and, at the same time, the accumulation of waste. A large number of unused drugs are thrown away or destroyed. The expiration of the shelf life is another reason for disposal of drugs.

The very high cost of disposing or storing expired drugs, estimated to be billions of dollars ^(1)^, has motivated scientists to focus on the issue and a number of studies on expired drugs have been conducted to test the safety and efficacy of these expired drugs on patients ^(2)^.

## The Expiry Date and Shelf Life

The expiry date is the date at which the manufacturer can still guarantee the full potency and safety of the drug at a specific point in time ^(3), (4)^. The term “shelf life” of a drug has a slightly different meaning; it is the period of time during which a drug remains safe for use. Most drugs expire after two or three years, and the manufacturer does not recommend using them after expiration. Other drugs have been found to have more than 5 years expiry date and this has been justified by independent laboratories. However, most of the expiration dates are set by the manufacturers themselves and regulatory bodies are not involved in the process. The manufacturer’s involvement is only for a limited number of carefully selected drugs ^(5)^.

The concept of shelf life started more than 50 years ago (1979) when the health authority in the United States, the Food and Drug Administration (FDA), asked manufacturers to set a limitation date for use for pharmaceutical products to ensure they work safely and effectively for patients. To determine the shelf life of a new product, the manufacturer conducts stability studies in specific environmental conditions to analyze any change in the quality of the product. The guidelines consist of accelerated stability study at a temperature of 40°C and 75% humidity for 6 months to understand how the product degrades under stress and real time (also called long-term) study at normal conditions of 30°C/65% for minimum 24 months to establish a tentative expiry date ^(6)^. The study is also important to determine optimal storage conditions, retest period and ensure the overall quality of a pharmaceutical product.

Since then, all pharmaceutical and medical products are required to have an expiry date in order to be released on the market, i.e., to obtain the marketing authorization approval and be available for patients.

According to the European Medicines Agency’s (EMA) guidelines on stability testing it is easy to shorten the expiration date, but a manufacturer needs to provide additional supportive data to extend the retest period beyond the end of the real time studies ^(7)^. With this in mind, a variation on the stability data for a marketing authorization is possible.

## Many Pharmaceutical Products Are Durable after Expiry of the Shelf Life

The term “expiration date” is sometimes mistermed, the dates on drugs labels are the point up to which pharmaceutical companies guarantee their safety but it does not necessarily mean they are not effective immediately after they expire, but instead that there are no data or studies to demonstrate they could still be used beyond that point. In fact, if they are stored and handled properly, many products have a high potency long after the expiration date ^(8)^. For example, a study reported by the Journal of Pharmaceutical Sciences in 2006 found that 2/3 of 122 expired pharmaceutical products tested for efficacy were still stable and safe for use for months. Thus, the expiration date was extended by one to five years for some of them, with an average extension of 66 months ^(9)^. Many other medicines, if stored properly, can retain more than 70% of their potency for years, although the container has been opened ^(10)^. However, this is not systematic for all products; for example, nitroglycerin, insulin, epinephrine, tetracycline and some antibiotics are not recommended for use after expiry. It is further advised for the patient to dispose of them safely, ideally by returning to the pharmacy or hospital, rather than throwing them away or flushing them down the drain. The latter has been proven to be harmful for the environment ^(11)^.

## The Regulation of Dispensing Expired Drugs

The regulations and legislation in most countries prohibit pharmacists and healthcare providers from dispensing expired drugs ^(12)^. In 2019, it was reported that one pharmacy received a reprimand from the North Carolina Board of Pharmacy for dispensing expired medication to patients ^(13)^.

Many countries have special guidelines to properly dispose of expired pharmaceutical products in pharmacies, hospitals and households ^(14)^, but at a global level, there is no harmonized approach to monitor and control such products. Handling and implementation of recommendation is different from one country to another. The European Union also has guidelines for safe disposal but there is no clear coordination between the private and government sectors. On a global level, the World Health Organization guidelines are: 1. return to donor or manufacturer, 2. high temperature incineration, 3. immobilization by waste encapsulation, 4. chemical decomposition.

There is currently no regulation on how long a pharmaceutical product should last; the standard of 2 to 5 years has been set rather through practice. Given the interest it represents, some organizations are in a favor of reviewing this policy, for example the Comité Permanent des Médecins Européens (CPME) calls upon European Commission to revise the rule of possibility of extending expiration dates for pharmaceuticals ^(15)^.

In summary, there is an urgent need for a globally harmonized and comprehensive approach for all stakeholders to monitor and control expired pharmaceuticals, and also for the disposal of expired pharmaceuticals.

## The Advantages of Using Expired Pharmaceuticals

The loss of expired medication has several disadvantages. Many developing countries and countries with middle income cannot afford pharmaceuticals and they suffer from an acute shortage in many vital drugs such as antibiotics and some others. It would be more ethical to redirect expired medication, proved to be safe for use, to those countries, avoiding waste and saving money. Potentially it is possible to use expired medication by the donor and to provide fresh batches to countries in shortage or in need of medication, to show good will and solidarity. Therefore, extending the shelf life by months or years can make these drugs more accessible and affordable to patients with low incomes. These drugs offer a hope for these populations. At the same time, health authorities and insurance companies can take advantage because this helps to save money and to reduce shortage of some medications. However, expired drugs, proven to be safe and efficient, could be particularly helpful in case of emergency need as they can be delivered faster than producing new batches.

## The Financial Interests of Pharmaceutical Companies

Most manufacturers know that the shelf life of products they produce has a longer period and the expiry date can be extended by months or years. This could be done if they conducted further studies, but they do not take any action. They take advantage of the absence of regulation or of a law on the shelf life extension, so they do not feel obliged to make such efforts; they can also claim that they do not want to risk patient safety, when it is quite possible that safety and efficacy would not in fact be compromised.

Conducting extension studies has a cost and is a time-consuming procedure so, there is no economic interest for them to investigate further. The cost for long- or short-term stability may not be so expensive, however, some other additional factors may increase the expenses such as logistic, human, regulatory and time. Where it is in their interest when medications are tossed as “expired” despite their safety and efficacy, fundamentally, pharmaceutical companies have a conflict of interest that biases them against taking steps toward extending the usable life of drugs; if a drug is thrown away, a replacement product will have to be bought.

Many pharmaceutical companies prefer to focus on sales and use the profit to develop more products. Making it a barrier for research and development activities, the obligation of the extension of expiry date may have negative results on their performance and on the pharmaceutical industry in general. This can also reduce access and increase in the price of pharmaceutical products.

## The Solution of the Shelf Life Extension Program

In the late 1980’s, the FDA and the United States Department of Defense (DoD) agreed to extend the expiration date of certain drugs in order to save replacement cost, further to a request by the Air Force. Thus, the Shelf Life Extension Program (SLEP) was created for the first time ^(16)^.

Each year, drugs from stockpiles are selected based on their value and pending expiration, and analyzed in batches to determine whether their end dates could be safely extended. Experience has found that the real shelf life of many drugs is well beyond the original expiration date. No one has been harmed by any expired drugs in the study after use.

In some cases, including stockpiler-initiated, the FDA may allow the Emergency Use Authorisation (EUA) in case an expired product is considered unapproved, but this is limited to Chemical, Biological, Radiological, Nuclear and Explosive (CBRN) emergencies.

## Analysis

The debate is ongoing about the dilemma of expired medication. Should we give priority to the patients’ health, or should we support the pharmaceutical industry that is a major player in making medications available for us. Although industry might argue that it would not be in patient’s interests to extend the life of drugs, it is clearly in the interest of patients to let them access affordable medicines by giving a second life for expired ones. This could also save healthcare systems money and reduce insurance premiums by reducing the amount of money spent on unnecessary replacement for jettisoned pharmaceuticals.

The important question for regulators is how, taking into consideration the interests of pharmaceutical manufacturers, to implement a policy to establish more accurate expiration date labeling. One option is to require all pharmaceutical companies to complete long-term stability testing. Running an extension program has a cost but it obviously helps to save much more from replacing expired drugs.

Some people are unable to afford such expensive medication; the extension of the shelf life of expired products can save them. Longer shelf lives could also play a role in decreasing medication shortage. The SLEP gives a second life to expired products and besides its ethical and therapeutic advantages also offers financial convenience.

Obviously, SLEP and EUA have benefits to the healthcare system and patient more than to pharmaceutical companies. Damages incurred by companies could be on many levels. First, by extending the shelf life of existing medication, financial manufacturers will not produce such medication for a while, and this is a shortfall for their business.

In the long term, if the manufacturers see the extension applied often to a specific medication, they may stop producing it owing to no interest.

On a regulatory level, if the manufacturer stops producing for a long time, he or she may lose the product license.

Also on an employment level, some positions could become redundant and there could be a reputational loss.

Most people think that drugs are not safe or efficient to use after the expiry date and this is also the thinking of many healthcare providers who are influenced by the claim of the drug manufacturers. There are many programs to extend the shelf life of expired drugs around the world, but they are not systematic, and mostly are case to case and sometimes batch to batch ^(17)^.

## Conclusion

Several studies have shown additional evidence on the full potency of pharmaceuticals beyond their expiry date, sometimes for decades. The extension of the shelf life is a good solution to give a second life for these products, to avoid the shortage of some medications and to make drugs affordable for people with low income. In fact, extending the safe efficacious life of drugs is an ethical imperative.

Nowadays most expired drugs are forgotten for different reasons, whether intentionally or unintentionally. If the product is proven to be safe and efficient for use, it will be helpful to put it again in the market and make it available for most needed patients. But also, the concept should not harm the pharmaceutical industry and slow their ability to bring us new and improved formulation.

## Article Information

### Conflicts of Interest

None

### Author Contributions

The authors confirm contribution to the paper as follows: Anis Arioua; data collection, analysis and interpretation of results, draft manuscript preparation. David Shaw: reviewed and advised on the final version of the manuscript.
